# Evaluation of the Human Health Risk of Wild Edible Mushrooms Consumption from Batak Mountain, Bulgaria

**DOI:** 10.18502/ijph.v49i8.3906

**Published:** 2020-08

**Authors:** Miroslava IVANOVA, Lilko DOSPATLIEV, Denitsa GRIGOROVA, Penko PAPAZOV

**Affiliations:** 1. Department of Informatics and Mathematics, Trakia University, 6000 Stara Zagora, Bulgaria; 2. Department of Pharmacology, Animal Physiology and Physiological Chemistry, Trakia University, 6000 Stara Zagora, Bulgaria; 3. Department of Probability, Operations Research and Statistics, Sofia University “St. Kliment Ohridski”, 1164 Sofia, Bulgaria; 4. Department of Organic Chemistry and Inorganic Chemistry, University of Food Technology, 4000 Plovdiv, Bulgaria

## Dear Editor-in-Chief

Currently, 14000 mushroom species are known to exist (1). This study aimed to determine Pb, Cd, Ni, Cr, Mn, Co, Cu and Zn contents in 9 species wild edible mushrooms growing in the Batak Mountain, Bulgaria and thus to assess the health risk index arisen from the long-term consumption of them.

Mushroom samples were collected in 2014 and 2018 from the Batak Mountain, Bulgaria. The Batak Mountain is located in western Rhodopes, Bulgaria (GPS41°46′02.6″N 24°08′48.4″E). The region is industry-free and is characterized by forests, land and low buildings. R 3.4.4 program for Windows was used for statistical data processing. Quantitative determination of the concentration of the studied elements was carried out in the mineralized samples by ICP Optima model 7000 DV. The daily intake of metals (DIM) was determined (2).

The average BW was taken as 70 kg for adults and 61, 43, 23, 12 kg for children: 14–18, 10–14, 3–10, 1–3 yr old. The health risk index (HRI) for the local population through the consumption of mushrooms was assessed using (3). HRI of <1 means the exposed population was assumed to be safe.

For the age group 1–3 years the HRI values were bigger than 1 for the following mushrooms *Amanita caesarea* (1.4638), *Cantharellus aurora* (1.3545) and *C. tubaeformis* (1.1839). The quantity of 0.071 g fresh weight was too high for this age group and the daily intake should be half of it at most. Mushroom types *A. caesarea*, *C. aurora* and *C. tubaeformis* had the most highest percentage content of Cu from HRI (46.46%, 29.32% and 32.54%).

The second element with the highest percentage content from HRI was Cd (33.47%, 24.77% and 23.84%). For the mushroom types *Lactarius deliciosus* and *Tricholoma equestre* the element Cd had the highest percentage content from HRI (35.09%, 72.14%) and the element with the smallest percentage content was Co (0.25%, 0.23%). The elements Cd and Cu (35.60%, 34.54%) had highest percentage content from HRI for the mushroom *Boletus pinophilus*, while the element Co had the smallest percentage content (0.35%). The element Cu (36.95%) had the highest percentage content from HRI for the mushroom *C. cibarius*, while the element Ni (1.45%) had the smallest percentage content. The element Cr (37.92%) had the highest percentage content from HRI for the mushroom *Craterellus cornucopioides*, while the element Ni (3.44%) had smallest percentage content. The element Cd (38.45%) had the highest percentage content from HRI for the mushroom *Morchella esculenta*, while the element Zn (0.96%) had the smallest percentage content. The concentration of Cd in the mushrooms were under the threshold according to Commission Regulation № 629/2008 (4).

**Fig. 1:**
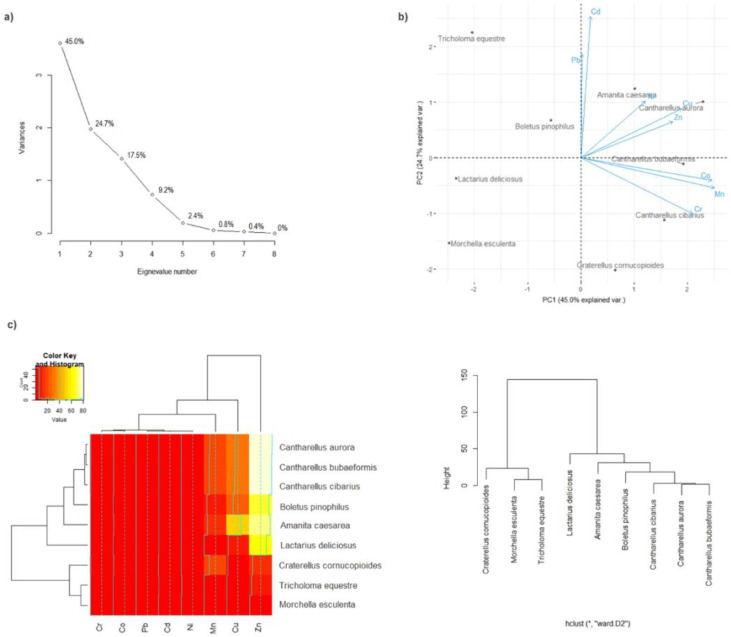
PCA of 9 species wild edible mushrooms: a) Eigenvalue of the correlation matrix; b) PCA of mushrooms based on their trace elements; c) Heatmap with presentation of a hierarchical tree plot to show the groups of mushrooms characterized by a high similarity to all element accumulation

Principal component analysis (PCA) was used to demonstrate similarities and differences in the accumulation of 8 trace elements in 9 species wild edible mushrooms. From the screen plot graph of eigenvalues of the PCA (Fig. 1a) we may notice that first two PCs are enough to explain 69.7% of total variability of mushrooms to the observed element accumulation (5). The first principal compound (PC1) explained 45% of the variation, while the second principal component (PC2) contributed 24.7% of the total variation. Graphical representation of the mushroom species in the space of the first two components of the performed PCA is presented in Fig. 1b. Accumulation of Co, Cr, and Mn was independent of Cu, Ni, and Zn as well as Cd and Pb. Simultaneously two groups of elements predominantly influenced element accumulation in individual mushroom species, i.e. the following relationships were observed: *C. tubaeformis* and *C. cibarius* about Co, Cr, and Mn; *A. caesarea* and *C. aurora* - Cu, Ni, and Zn.

To show the similarities or differences with regard to all 8 elements contents between mushroom species, the obtained results were illustrated using a Heatmap analysis, where two-dimension variables were represented by colors. Cluster Analysis allowed the selection of mushroom species and analyzed elements in the way that the relation between observations inside the same group was shown to be possibly the highest, while between different groups it was the lowest. Using Ward Hierarchical Clustering and Euclidean distances, dendrogram showing clustering was obtained. The first cluster contains *C. cornucopioides*, *M. esculenta* and *T. equestre* while the second cluster contains *Lactarius deliciosus*, *A. caesarea*, *Boletus pinophilus*, *C. cibarius*, *C. aurora* and *C. tubaeformis* (Fig. 1c).
